# Anticancer Effect of Fucoidan in Combination with Tyrosine Kinase Inhibitor Lapatinib

**DOI:** 10.1155/2014/865375

**Published:** 2014-01-22

**Authors:** Byeongsang Oh, Jihun Kim, Weidong Lu, David Rosenthal

**Affiliations:** ^1^Dana-Faber Cancer Institute, Harvard Medical School, Boston, MA 02215, USA; ^2^Sydney Medical School, University of Sydney, Sydney, NSW 2006, Australia; ^3^University of Ulsan College of Medicine, Asan Medical Center, Seoul, Republic of Korea

## Abstract

*Background*. Despite a number of *in vitro* and *in vivo* studies reporting the efficacy of fucoidan in treating various cancers, few studies have measured the efficacy of dietary fucoidan (DF) in combination with cancer drugs. Thus, we examined the sensitivity of DF in combination with the EGFR/ERBB2-targeting reagent lapatinib on cancer cells. *Method*. We selected six EGFR/ERBB2-amplified cancer cell lines (OE19, NCI-N87, OE33, ESO26, MKN7, and BT474) as an *in vitro* model and tested their sensitivity to DF alone and to DF in combination with the well-known EGFR/ERBB2-targeting reagent lapatinib. *Result*. Overall, in drug independent sensitivity test, DF alone did not significantly inhibit the growth of EGFR/ERBB2-amplified cancer cells *in vitro*. When DF was given in combination with lapatinib, however, it tended to synergistically inhibit cell growth in OE33 but antagonized the action of lapatinib in ESO26, NCI-N87, and OE19. *Conclusion*. This study suggests that DF has the potential to increase or decrease the effects of certain anticancer drugs on certain cancer cell types. Further study is needed to explore the mechanism of interaction and synergistic antitumor activity of DF in combination with chemotherapy and targeted therapy.

## 1. Introduction 

The use of dietary supplements (DS) is gaining in global popularity as a form of complementary and alternative medicine (CAM) [1, 2]. DS, defined as any product that contains vitamins, minerals, herbs or other botanicals, amino acids, enzymes, and/or other ingredients intended to supplement diet, is currently one of the most increasingly used CAM therapies [3]. A study found that 69% of the cancer patients in the US use DS following their cancer diagnosis [4]. A recent Australian survey with cancer patients also reported that approximately 69% of respondents had used one form of CAM in the previous year and 41% of them had visited a CAM practitioner [5]. Currently, thousands of DS products are available over the counter without health professionals' prescription, though the majority of DS have yet to be evaluated in clinical trials. Thus, most oncologists are concerned about possible herb-drug interactions that might occur with conventional anticancer drugs resulting in either excess toxicity or reduced efficacy [6]. A few studies have examined the interaction between herbs and chemotherapy drugs, but none have been examined yet to look at targeted agents [6]. And so, study on DS is essential to provide evidence-based information for cancer patients as well as healthcare practitioners.

Fucoidan, one kind of DS, has been reported to exhibit anti-inflammatory, antibacterial, antiviral, immunomodulating, antiangiogenesis, and antitumor activities [7–10]. Fucoidan, a sulfated polysaccharide found in cell walls of various species of brown and green seaweeds, is known to contain large proportions of L-fucose and sulfate along with low amounts of xylose, uronic acid, and galactose [9, 11]. Seaweed and marine algae have been used as food and medicine for over a thousand years in Asia and in some parts of northern Europe [12]. Recently, the antitumor effect of fucoidan has been studied intensively. Studies have reported that the intake of seaweed is associated with a lower incidence of breast cancer [13, 14].

A recent study of patients with unresectable advanced or recurrent colorectal cancer suggests that fucoidan has the potential to reduce the toxicities of chemotherapy [15]. *In vivo* studies conducted using mouse xenograft models have suggested that fucoidan suppresses the growth of 4T1-derived breast cancer [16], A20-derived lymphoma [17], and Ehrlich ascites carcinoma [18], inhibits metastasis of Lewis lung adenocarcinoma [19] and 13762 MAT rat mammary adenocarcinoma, [20] and has antiangiogenesis activity against B16 melanoma [21]. The lines of evidence of *in vitro* studies have demonstrated that fucoidan inhibits the growth of non-small cell bronchopulmonary carcinoma NSCLCN6 cells [22] and human lymphoma HS-Sultan cells [23] and also induces apoptosis in cells derived from human lymphoma [23], promyelocytic leukemia [24], colon carcinoma [7], breast carcinoma [16], ovarian carcinoma, and hepatoma [25], including the prevention of angiogenesis by suppressing expression and secretion of the angiogenesis factor, vascular endothelial growth factor (VEGF) [26]. However, the mechanism involved in the anticancer action of fucoidan is incompletely understood, and further studies on fucoidan and chemotherapy interactions are limited.

Lapatinib, a targeted therapy that acts as a tyrosine kinase inhibitor, is extensively used to treat advanced HER2-positive breast cancer as well as other cancers [27]. Tyrosine kinases are plasma membrane proteins that signal cells to divide and grow. Lapatinib blocks signals from these proteins which help to slow cancer cell growth [27]. In particular, lapatinib interferes with protein tyrosine kinases, epidermal growth factor receptor type 1 (EGFR (ERBB1)), and human epidermal receptor type 2 (HER (ERBB2)). Lapatinib, administered orally as active small molecules, interferes with the growth of cancer cells and potentially with the growth of normal cells.

Cancer patients receiving targeted therapy lapatinib may have used fucoidan as a DS, although drug interactions of fucoidan-anticancer targeted therapy have not been studied. Hence, in our current study, we examined the sensitivity of OE19, OE33, ESO26, NCI-N87, BT474, and MKN7 cancer cells to DF and to DF in combination with lapatinib.

## 2. Material and Method

### 2.1. Cell Lines and Reagents

OE19, NCI-N87, OE33, and ESO26 cells were obtained from the American Type Culture Collection (ATCC) and MKN7 cells were obtained from the Japanese Cell Resource Bank (JCRB). BT474 cells were kindly provided by the Hahn Laboratory. Lapatinib was purchased from LC Laboratories and was dissolved in dimethylsulfoxide (DMSO) (Sigma-Aldrich, St. Louis, MO). Samples of dietary fucoidan extract were obtained from EnzAlg Bio Sciences (New Zealand) and were dissolved in phosphate-buffered saline (PBS).

### 2.2. Cell Culture

All cell lines were maintained by RPMI-1640 medium (Mediatech, Manassas, VA) supplemented with 10% Fetal Bovine Serum (Gemini Bio-Products, West Sacramento, CA) and were kept in a humidified incubator at 5% CO2. For all cell lines, the passage number was carefully controlled and mycoplasma contamination was monitored on a regular basis.

### 2.3. Drug Sensitivity Assay

For a drug sensitivity assay, we used Cell-Titer Glo cell viability luminescence assay (Promega). Briefly, cells were plated at a desired density (OE19, 2 × 10^3^; OE33 and MKN7, 1 × 10^3^; NCI-N87, 3 × 10^3^; ESO26 and BT474, 5 × 10^3^) in 96-well plates. After 24 hours, cells were treated with variable doses of lapatinib or fucoidan extract alone or in combination and then cultured for 72 hours. In the control, cells were treated with DMSO for lapatinib and PBS for fucoidan extract. At the end of the experiment, the number of viable cells was estimated using the Cell-Titer Glo luminescence assay according to the manufacturer's instructions. All experiments were repeated three times with each biological replicate including [4] replicates per treatment condition.

### 2.4. Statistical Method

A dose-inhibition curve was drawn from the results of the drug sensitivity assay by calculating percent inhibition (100 × cell growth rates of drug-treated wells/growth rates of vehicle-treated wells) and log-transforming the drug doses. IC_50_ values were estimated based on the curves obtained. In the herb-drug combination experiments, the statistical significances of the differences in cell growth were estimated by one-way ANOVA. All statistical analyses were done using GraphPad Prism software (Ver 6.0a).

## 3. Results

### 3.1. Various ERBB2-Amplified Gastroesophageal and Breast Cancer Cell Lines Show Different Sensitivities to Lapatinib

**Figure 1:**
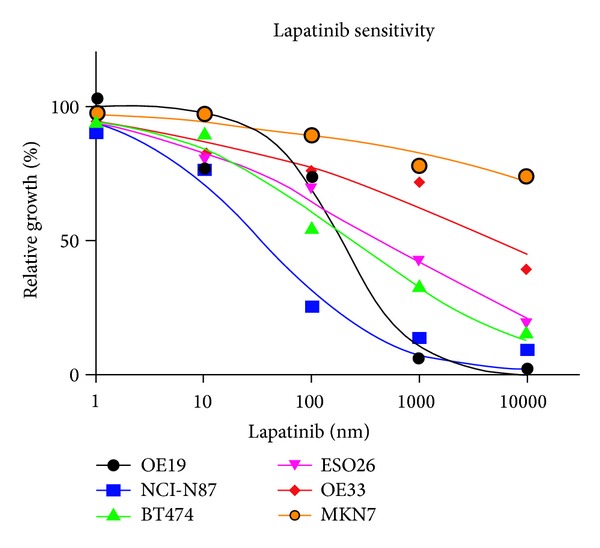


ERBB2 amplification has been known to predict the sensitivity to the EGFR/ERBB2-targeted small molecule inhibitor, lapatinib [27]. However, the *in vitro* drug sensitivity assay performed in this study revealed variable drug sensitivity profiles among the different ERBB2-amplified cell lines (details regarding differential sensitivity of cell lines to lapatinib are being reported separately (manuscript in preparation)). OE19, NCI-N87, and BT474 cells were sensitive to lapatinib whereas OE33 and MKN7 cells were resistant to lapatinib. ESO26 cells were moderately sensitive to lapatinib (Figure 1). IC_50_ values are summarized in Table 1.

### 3.2. Fucoidan Extract Does Not Have Significant *In Vitro* Growth Inhibitory Effect on Various ERBB2-Amplified Gastroesophageal and Breast Cancer Cell Lines

**Figure 2:**
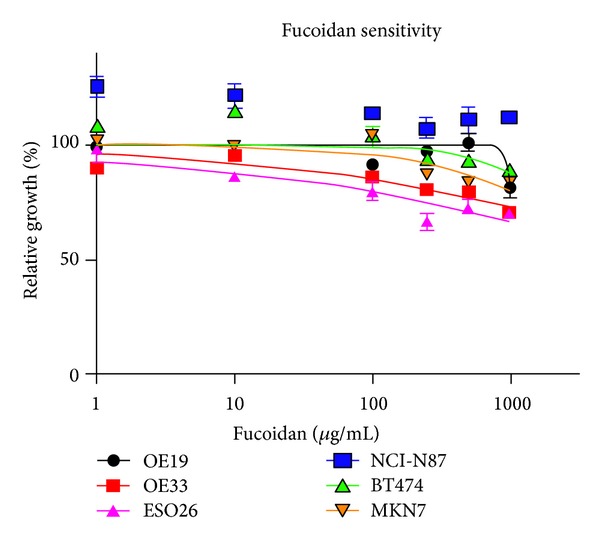


Overall, in dose-dependent drug sensitivity test, DF, as a single agent, did not significantly inhibit the growth of EGFR/ERBB2-amplified gastroesophageal and breast cancer cell lines even at supra-pharmacological doses (Figure 2). Only OE33 and ESO26 cells showed equivocal trends of dose-dependent growth inhibition.

### 3.3. Fucoidan Extract, When Combined with Lapatinib, Has Variable Effects on Cancer Cell Growth in Different Cell Lines

**Figure 3:**
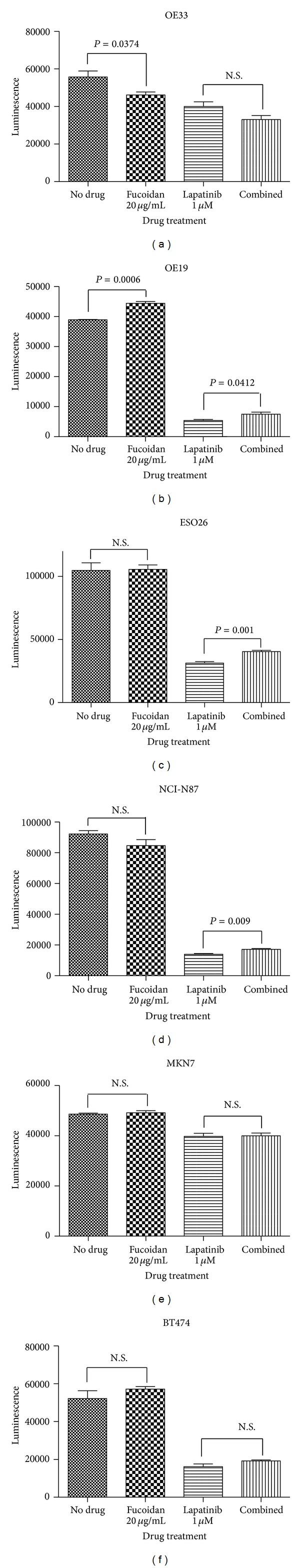


Treatment with 20 *μ*g/mL of DF has a less significant effect on inhibition of OE33 cell growth than that of 1 *μ*M of lapatinib. When DF was combined with lapatinib, it tended to be more effective on the inhibition of OE33 cell than lapatinib alone, but the difference was not significant (Figure 3(a)). However, DF treatment with 20 *μ*g/mL increased the growth of OE19 cells as well as DF in combination with lapatinib (Figure 3(b)). In ESO26 and NCI-N87 cells, DF had antagonistic effects on cell growth when combined with lapatinib, while DF alone had no significant effect (Figures 3(c) and 3(d)). In MKN7 and BT474 cells, DF had no effect on cell growth either as a single agent or in combination with lapatinib (Figures 3(e) and 3(f)).

## 4. Discussion 

Dietary supplementation is most commonly used by cancer patients even though there is limited evidence on its efficacy in this patient population. In the last decade, numerous *in vivo* and *in vitro* studies have suggested that fucoidan, extracted from seaweed, has an anticancer effect. No published randomized controlled trial (RCT) has assessed the effect of fucoidan when combined with targeted therapy pharmacokinetics, despite the fact that no adverse reactions had been reported from the use of fucoidan [28]. Thus, to investigate a fucoidan-drug interaction prior to a design of a future clinical trial, we examined the sensitivity of DF alone and in combination of lapatinib (kinase inhibitor) on OE19, OE33, ESO26, NCI-N87, BT474, and MKN7 cancer cells. Our study found that DF extract alone had no significant growth inhibitory effect on various EGFR/ERBB2-amplified gastroesophageal and breast cancer cell lines *in vitro*. However, it did exhibit a synergistic effect on OE33 when combined with lapatinib, while exhibiting an antagonistic effect on NCI-N87, ESO26, and OE19 cancer cells. These results suggest that the effect of DF plus lapatinib may be cell type dependent.

Recently, two studies have reported the effect of fucoidan on cancer when administered in combination with cancer drugs. Zhang et al. examined the effect of fucoidan plus chemotherapy agents (cisplatin, tamoxifen, or paclitaxel) on breast cancer cells (MDA-MB-231 and MCF-7). The results demonstrated that fucoidan in combination with cisplatin, tamoxifen, or paclitaxel significantly enhanced cell death of MDA-MB-231 and MCF-7 breast cancer cells by regulating the expression of Bcl-2 family proteins, modulating ERK and Akt signaling, and regulating the production of oxidative stress [29]. Our current study suggests that fucoidan plus the targeted cancer drug lapatinib can have either an synergistic or antagonistic effect, depending on the type of cancer cell treated. Although we did not experiment on the same cancer cell types as those used in the Zhang et al. study, our results are inconsistent with the findings in the Zhang et al. study. In the second recent study, Ikeguchi et al. conducted a clinical trial on unresectable advanced or recurrent colorectal cancer patients (*n* = 20) to investigate the effect of fucoidan in combination with chemotherapy (oxaliplatin plus 5-fluorouracil/leucovorin (FOLFOX) or irinotecan plus 5-fluorouracil/leucovorin (FOLFIRI)) and reported that fucoidan plus FOLFOX or FOLFIRI has the potential to reduce the toxicity and improve survival by prolonging the duration of clinical effectiveness of chemotherapy [15]. As a result, the need for further studies on the application of fucoidan in combination with chemo- and/or targeted drug approaches was suggested to address management of toxicity, clinical outcome, and mechanism of herb-drug interaction.

A major limitation of this study is that experiments were performed *in vitro*. However, to our knowledge, this is the first study that has examined and demonstrated a potential DF-targeted therapy lapatinib interaction. Clinical trials and animal testing are needed to confirm the results of our study as the next step towards clinical application. Another limitation is that the quality of DF product (e.g., structure, composition, or molecular weight) was not evaluated. The study was designed to examine the anti-cancer effects on cancer cells with DF available to cancer patients as a commercial over-the-counter product available without doctor's prescription.

Recent literature reviews have suggested that a major concern among doctors is the use of CAM before and during cancer treatment due to potential herb-drug interactions reducing the efficacy of cancer treatment or producing adverse effects. Azuma et al. suggested that, regardless of molecular weight (lower-molecular-weight fucoidan (LMWF: 6.6–40 kDa), intermediate-molecular-weight fucoidan (IMWF: 110–138 kDa), and high-molecular-weight fucoidan (HMWF: 300–330 kDa)), fucoidan-containing diet increases the survival time of mice [30]. Still, Matsubara et al. argue that the pharmacological effects of fucoidan vary with molecular weight [31]. Hence, further studies are required to determine if molecular weight of fucoidan is associated with the inhibition of tumor growth and an increase in the efficacy of targeted drugs. It will also be beneficial to test the efficacy of DF in combination with multiple cancer drugs on various cancer cells to establish herb-drug interaction guidelines and to discover potential new drugs that can enhance survival of cancer patients. Furthermore, our present study did not examine the mechanism of fucoidan-lapatinib interaction. Further *in vivo* and clinical studies are required to address a potential fucoidan-drug interaction.

In conclusion, co-administration of DF and lapatinib in cancer patients produces a potential herb-drug interaction. An animal study and clinical trial are necessary to evaluate the pharmacodynamic impact of fucoidan on molecularly targeted therapy and chemotherapy in cancer treatment.

## Figures and Tables

**Table 1:** IC_50_ values of the various ERBB2-amplified gastroesophageal and breast cancer cells treated with lapatinib.

Cell lines	IC_50_ (nM)
OE19	188
NCI-N87	34
BT474	236
ESO26	433
OE33	>1,000
MKN7	>1,000
